# Modelling heterogeneity in malaria transmission using large sparse spatio-temporal entomological data

**DOI:** 10.3402/gha.v7.22682

**Published:** 2014-06-24

**Authors:** Susan Fred Rumisha, Thomas Smith, Salim Abdulla, Honorath Masanja, Penelope Vounatsou

**Affiliations:** 1 Department of Epidemiology and Public Health, Swiss Tropical and Public Health Institute, Basel, Switzerland; 2 Department Biozentrum, University of Basel, Basel, Switzerland; 3 Department of Disease Surveillance and Geographical Information Systems, National Institute for Medical Research, Dar es Salaam, Tanzania; 4 Ifakara Health Institute, Dar es Salaam, Tanzania

**Keywords:** approximate spatial process, malaria transmission, seasonality, MCMC, INDEPTH-MTIMBA

## Abstract

**Background:**

Malaria transmission is measured using entomological inoculation rate (EIR), number of infective mosquito bites/person/unit time. Understanding heterogeneity of malaria transmission has been difficult due to a lack of appropriate data. A comprehensive entomological database compiled by the Malaria Transmission Intensity and Mortality Burden across Africa (MTIMBA) project (2001–2004) at several sites is the most suitable dataset for studying malaria transmission–mortality relations. The data are sparse and large, with small-scale spatial–temporal variation.

**Objective:**

This work demonstrates a rigorous approach for analysing large and highly variable entomological data for the study of malaria transmission heterogeneity, measured by EIR, within the Rufiji Demographic Surveillance System (DSS), MTIMBA project site in Tanzania.

**Design:**

Bayesian geostatistical binomial and negative binomial models with zero inflation were fitted for sporozoite rates (SRs) and mosquito density, respectively. The spatial process was approximated from a subset of locations. The models were adjusted for environmental effects, seasonality and temporal correlations and assessed based on their predictive ability. EIR was calculated using model-based predictions of SR and density.

**Results:**

Malaria transmission was mostly influenced by rain and temperature, which significantly reduces the probability of observing zero mosquitoes. High transmission was observed at the onset of heavy rains. Transmission intensity reduced significantly during Year 2 and 3, contrary to the Year 1, pronouncing high seasonality and spatial variability. The southern part of the DSS showed high transmission throughout the years. A spatial shift of transmission intensity was observed where an increase in households with very low transmission intensity and significant reduction of locations with high transmission were observed over time. Over 68 and 85% of the locations selected for validation for SR and density, respectively, were correctly predicted within 95% credible interval indicating good performance of the models.

**Conclusion:**

Methodology introduced here has the potential for efficient assessment of the contribution of malaria transmission in mortality and monitoring performance of control and intervention strategies.

Malaria is still endemic in more than 100 countries worldwide, leaving children and pregnant mothers being the most vulnerable groups for infections (1). Global estimates report 219 million malaria cases (range 154–289 million) with about 660 thousands deaths (range 610–971), most of these (~90%) occurring in Africa. The impact of the malaria burden on the achievement of Millennium Development Goals is enormous, and its control is a potential contribution towards significant progress (1).

Malaria is transmitted by female *Anopheles* mosquitoes. The transmission intensity is therefore highly sensitive to environmental variations that affect the densities of these vectors and their ability to transmit the infection (2–4). Up to 10-fold variations in transmission intensity have been observed within very small localities due to geographical, biological or socio-economic factors (5–8). Understanding the heterogeneity in transmission and human exposure to malaria infection is critical for optimizing control programs and targeting interventions (9–12).

Malaria disease burden and transmission can be assessed using incidence or prevalence in human hosts. However, the entomological inoculation rate (EIR) most directly quantifies the exposure of the human population to the infectious stages of the parasite (12–16). EIR is the product of the human-biting rate, for example, mosquito bites/person/night (which can also be estimated using mosquito density) and the sporozoite rate (SR), which is the proportion of infective mosquitoes (7, 17). The measure expresses the average number of infective bites a person receives in a specified unit of time. It can be also used to predict other measures of transmission, which are used to evaluate effectiveness of malaria control program (5, 18). Uncertainty due to small sample, low values and variability in the SR and cost complicate precise estimation of EIR requiring standardized entomological surveys conducted over large areas (5, 6, 13, 14). Accurate estimation of EIR requires longitudinal surveys within the study area to take into account spatio-temporal variations and seasonality trends. However, there is a paucity of this type of data due to cost and resources needed to collect them (19–21).

The Malaria Transmission Intensity and Mortality Burden across Africa (MTIMBA) project was initiated by the INDEPTH Network (22, 23) and conducted over a period of 2001–2004 in several countries in Africa including Tanzania, Kenya, Mozambique, Senegal, Ghana and Burkina Faso. The main objective of the initiative was to assess the relation between the intensity of malaria transmission and all-cause as well as malaria-specific mortality across Africa, taking into account the influence of malaria control activities. The MTIMBA entomological data have been collected fortnightly over large number of locations (households) and to date this is the only available entomological database appropriate to study space–time heterogeneity of malaria transmission in Africa. These data are sparse with seasonal variations and spatio-temporal correlations. High dependence of climate, environment and ecological factors in the life of mosquito and seasonality any of the survey locations had zero mosquitoes or proportion of infected ones. In standard modelling approaches, EIR is treated as a continuous outcome, logarithmically transformed to fulfil the assumption of normality (21, 24–27). However, when EIR is estimated as a product of the SR and mosquito density, which are generated from the binomial and a count distribution like Poisson or negative binomial, respectively, normality assumptions are void. To our knowledge, Kasasa et al. (28) is the only literature report analysis of EIR data considering the two sources of data separately. In addition, due to the amount of zeros which is larger than what can be generated by the standard distributions, the data are over/under dispersed and zero inflated (21, 29–32). Statistical analysis which accounts for these characteristics is essential to obtain unbiased estimates for the regression coefficients (33–36).

Moreover, the MTIMBA-EIR data have been collected at fixed locations and they are typically geostatistical data. Similar exposures of environmental and climatic conditions to locations which are geographically close introduce spatial correlation between them. Geostatistical models take into account spatial correlation by introducing location-specific random effects as latent observations from a multivariate spatial Gaussian process (37). Spatial correlation between any pair of locations is often considered as a function of distance on the covariance matrix of the process. These models have a large number of parameters. Bayesian formulations (38) allow model fit via Markov Chain Monte Carlo (MCMC) simulation methods (39). However, the estimation process involves covariance matrix computations which are infeasible when the number of locations is too large (40, 41). A computational flexible way to overcome this problem is the approximation of the spatial process from a subset of locations using properties of conditional multivariate Gaussian distribution of the process (40–42). Most of these techniques have been applied in simulated data, observed in regular grid and mainly with Gaussian characteristics. In this study, selection of subset of locations is implemented using methods proposed in our previous work (40, 43).

We now demonstrate a rigorous modelling way of analysing large spatio-temporal EIR data and study the heterogeneity, space and temporal patterns of malaria transmission within one MTIMBA site, the Rufiji DSS area in Tanzania (44). The Gaussian process approximation proposed by Banerjee et al. (40) is applied to binomial (SRs) and negative binomial (density) data with zero inflation. The models are fitted using Bayesian MCMC simulation and assessed on the basis of their predictive ability. Model-based predictions of SR and density were multiplied to compute EIR. Model formulation details are given in the methodology section and selected results are presented afterwards. The discussion and conclusion of the findings consider the implications for timing and allocation of resources for malaria interventions.

## Methodology

### Study site

The study utilized data collected from one of the MTIMBA sites in Tanzania, the Rufiji DSS (RDSS). The RDSS is located in Rufiji District, Coast Region, Tanzania, about 178 km south of Dar es Salaam. The RDSS area extends from 7.47° to 8.03° south latitude and 38.62°–39.17° east longitude and operates in six contiguous wards and 31 villages. The surveillance area covers an area of 1,813 km^2^ and monitors 85,000 people, which is about 47% of the total population of the Rufiji District (INDEPTH Monogram). Rufiji District has an overall mean altitude of <500 metres. Its vegetation is mainly formed of tropical forests and grassland. The district has hot weather throughout the year and two rainy seasons: short rains (October–December) and long rains (February–May). The average annual precipitation in the district is between 800 and 1,000 mm. A prominent feature in the District is the Rufiji River with its large flood plain and delta, the most extensive in the country (INDEPTH Monogram; Rufiji DSS Profile, 2000). The majority of the people in the Rufiji District are subsistence farmers.

The main responsible malaria vectors in the area include *A. funestus*, and members of the *A. gambiae* complex, including *A. gambiae* (sensu stricto) and *A. arabiensis*. Mosquito populations usually peak during the rain seasons especially in areas where rice cultivation is taking place and during the dry months, a high population was usually observed in areas with permanent water bodies (23).

### Mosquito data

The entomological data were collected for the period of 3 years, October 2001–September 2004 (Source: http://www.indepth-network.org/dss_site_profiles/rufiji.pdf). The MTIMBA entomological protocol has been well defined in MTIMBA documentation (unpublished). In a snapshot, mosquitoes were captured at least twice every month using Centers for Disease Control (CDC) miniature light traps. The human population in the RDSS was classified into geographical clusters (100–1,000 people), then for each round a simple random sampling (without replacement) was employed within clusters to select between 20 and 100 ‘index’ people (households) for the set-up of mosquito catches (traps). The traps were fitted indoors with incandescent bulbs and laid close to a human volunteer (randomly selected from members of the household) sleeping under an untreated bednet. Light traps operated from sundown to sunrise (i.e. 6 pm–6 am) for two consecutive nights in each household and bags were emptied every morning. A total of 2,479 unique locations (households) involved were geo-referenced. Collected mosquitoes were counted and sorted into vector species to allow for separate assessment of transmission intensity.

### Environmental and climatic data

Remote sensing data were extracted from different sources with different spatial, Sp_R_, and temporal, T_R_, resolutions. These include normalized difference vegetation index (NDVI) (Sp_R_: 250 m^2^; T_R_: 16 days; Source: MODIS), day and night temperature (Sp_R_: 1 km^2^; T_R_: 8 days; Source: MODIS), rainfall (Sp_R_: 8 km^2^; T_R_: 10 days; Source: ADDS) and distance to the nearest water bodies (Sp_R_: 1 km^2^; Source: Health Mapper).

## Statistical analysis

Geostatistical zero inflated negative binomial and logistic regression models were fitted on the mosquito density and SR data, respectively. The models accounted for the effect of environmental and climatic predictors, annual trends, seasonal patterns, and spatial and temporal correlations. The predictive process was used to approximate the spatial process using a subset of locations. Model-based prediction of SR and density were multiplied to obtain estimates of monthly and annual EIR. Details of the model formulation and its implementation are described in the subsections below. Programs used for this analysis are available via contact with the corresponding author.

### Model formulation for density data

Let *Y*_*it*_ be the number of female mosquitoes and ${\text{X}}_{it}^{\left(1\right)}$ be a vector of environmental predictors (extracted from satellite data) observed at location ***s***_*i*_, ***i***=1,…,***n***, and calendar month *t*=1,…,36 for a specific species. *Y*_*it*_ is assumed to follow a negative binomial distribution, *Y*_*it*_~*NB*(*r*, *p*_*it*_), where *p*_*it*_=*r*/(*r*+*µ*_*it*_). ***r*** is an over-dispersion parameter and *µ*_*it*_ is the mean mosquito density. Covariates ${\text{X}}_{it}^{\left(1\right)}$, seasonal trends *f*(*t*)^(1)^, spatial ${\text{U}}_{i}^{\left(1\right)}$ , temporal $\varepsilon_{t}^{\left(1\right)}=(e_{1},e_{2},...,e_{t})$ and non-spatial ${\text{φ}}_{i}^{\left(1\right)}$ random effects are introduced on the log scale of the mean count via the equation $\log(\mu_{\text{it}})=X^{\text{T(1)}}\beta^{(1)}+f{\text{(t)}}^{(1)}+U_{i}^{\left(1\right)}+e_{t}^{\left(1\right)}+\varphi_{i}^{\left(1\right)}$ , where **β** ^(1)^ is the vector of regression coefficients, ${\text{φ}}_{i}^{\left(1\right)}$ is a residual error term capturing the remaining variability in the data. *f*(*t*)^(1)^ is modelled via trigonometric function with a mixture of cycle, *C*

$$f(t)=\sum_{c=1}^{C}\left\{\delta_{1c}^{\left(1\right)}*cos\left(\frac{2\pi }{T_{c}}t\right)+\delta_{2c}^{\left(1\right)}*sin\left(\frac{2\pi }{T_{c}}t\right)\right\},C=2;t=1,...,12/36$$

where *T*_*c*_ is the period of the season for cycle *C* (i.e. *T*_1_=12 and *T*_2_=6) and $\delta_{1c}^{\left(1\right)}$ and $\delta_{2c}^{\left(1\right)}$ are regression parameters used to describe the amplitude and phase within a period (45, 46). Separate models were fitted assuming: (i) a constant seasonal pattern across the 3 years of the study by taking *t*=1,…,12; or (ii) a continuous time for the entire study period by taking *t*=1,…,36. The seasonal pattern considering dry/wet categorization of the data was also assessed.

A zero inflated model formulation was adopted to take into account the excess zeros in the count data. The model is defined as a mixture of a degenerate distribution with mass at zero and a non-degenerate count distribution. The log-likelihood is therefore a sum of the log-likelihood for the non-zero and the zero counts. The distribution of the data is now defined as:

P(Y=0∣p*,θ)=p*+(1-p*)π(0∣θ)P(Y=y∣p*,θ)=(1-p*)π(y∣θ),y>0

where p* is the probability for a count to arise from the zero mass and 1–p* is the probability to observe a sample from a count distribution (i.e. π(*y*|θ)≡*NB* for our case, and θ is the vector of parameters associated with the distribution). This probability can be assigned a value between 0 and 1, usually approximates the proportion of zero counts in the sample or can be a function of covariates similar or different from those used in the full model (21, 33, 34, 47). Involving possible sources of zero inflation (e.g. covariates) reduces bias in parameter estimation of p* and other sources of uncertainty. In our case p* is modelled with a logit link as a function of all climatic predictors ${\text{X}}_{i}^{*}$ observed at location *s*_*i*_, i.e. $\log it({\text{p}}_{i}^{*})=X{*}^{T}\alpha$, where α is the corresponding vector of regression coefficients.

Bayesian model formulation requires the specification of prior distributions for all unknown parameters. For the regression coefficients, **β** ^(1)^, ***δ*** ^(1)^ and *α*, a standard non-informative uniform prior is adopted, i.e. **β** ^(1)^~Unif(–∞,∞), *δ* ^(1)^~Unif(–∞,∞) and ***α***~Unif(–∞,∞), respectively. The latent observations ${\text{U}}_{i}^{\left(1\right)}$ introduced at each location *s*_*i*_ are assumed to be derived from a multivariate normal distribution with a covariance matrix ${\text{Σ}}_{nxn}^{\left(1\right)}$ , i.e. $U^{\left(1\right)}\sim \text{MVN}(0,\Sigma_{nxn}^{\left(1\right)})$. The Σ^(1)^ is a matrix with elements $\Sigma_{ij}^{\left(1\right)}$ and quantify the covariance *Cov*(*U*_*i*_,*U*_*j*_) between the pair of locations *s*_*i*_ and *s*_*j*_, respectively. Its distribution defines the Gaussian spatial process. Under the assumption of stationarity, the spatial correlation is taken to be a function of distance between locations. An exponential correlation structure for the covariance matrix of the spatial process is adopted, that is $\Sigma_{ij}^{\left(1\right)}=\sigma_{sp}^{2^{\left(1\right)}}\exp(-d_{ij}\rho^{\left(1\right)})$, where $\sigma_{sp}^{2^{\left(1\right)}}$ is the spatial variance, *d*_*ij*_ is the distance between locations *s*_*i*_ and *s*_*j*_ and *ρ* ^(1)^ measuring the correlation decay and also known as the effective range (3/*ρ* ^(1)^) and estimates the distance where the spatial correlation is <5%. The decay parameter *ρ* ^(1)^ assumed to follow a gamma distribution.

Computation of the Gaussian process requires the inversion of the covariance matrix, Σ^(1)^, which for a very large number of locations is not feasible. To enable model fit we approximate the spatial process by a subset of locations, knots, {*s*_*i*_ *,*i*=1,…,*m*} (*m<<n*) with latent observations ***U*** ^*(*1*)^=(*U*(s_1_ *),…,*U*(s_m_ *))^*T*^. ***U*** ^*(*1*)^ is considered to arise from the same Gaussian process as ***U*** ^(*1*)^ and thus ***U*** ^*(*1*)^~*N*(0,Σ*), where Σ^*^ is the *mxm* covariance matrix of the sub-process. These latent observations ***U*** of the original process can be approximated by the ‘predictions’ of the sub-process via the mean of Gaussian conditional distribution $U^{\mathrm{(1)}}(s)|U^{{*}^{\mathrm{(1)}}}\sim N(Q^{T}\Sigma {*}^{-1}U^{{*}^{\mathrm{(1)}}},\sigma^{2}-Q^{T}\Sigma {*}^{-1}Q)$, that is $\hat{\text{U}}=Q^{T}\Sigma {*}^{-1}U^{{*}^{\mathrm{(1)}}}$, where $Q=Cov(U^{{*}^{\mathrm{(1)}}},U^{\mathrm{(1)}})$ is an *mxn* matrix of the covariance functions between the full and the sub-process (48, 49). Selection of subset of location was done using the minimax space filling design implemented in R software (50). The approach optimizes the selection of the best subset by minimizing the maximum of the nearest-neighbour distance between the original survey and the subset locations.

The ${\text{ε}}_{t}^{\left(1\right)}$ model temporal correlation via a stationary autoregressive process of order one, i.e. $e_{1}\sim Normal(0,\sigma_{T}^{2^{\left(1\right)}}/(1-\gamma_{}^{2}))$ and *e*_t_∣*e*_1,…*t*−1_~ *Normal* $e_{t}|e_{1,\ldots t-1}Normal(\gamma^{\left(1\right)}e_{t-1},\sigma_{T}^{2^{\left(1\right)}}),t\geq 2$, where $\gamma^{\left(1\right)}$ is an autocorrelation parameter $|\gamma^{\left(1\right)}|<1$ which adopts a bounded uniform distribution, $\gamma^{\left(1\right)}∼\mathrm{Unif}\left[-1,1\right]$ and $\sigma_{T}^{2^{\left(1\right)}}$ is the temporal error (51). The ${\text{φ}}_{i}^{\left(1\right)}$ is assumed to follow a normal distribution with mean zero and a homoscedastic variance $\sigma_{e}^{2^{\left(1\right)}}$. Inverse gamma priors are adopted for the variance parameters $\sigma_{sp}^{2^{\left(1\right)}}$, $\sigma_{T}^{2^{\left(1\right)}}$and $\sigma_{e}^{2^{\left(1\right)}}$.

### Model for SR

Let ***N***_*it*_ and ***Z***_*it*_ be the number of mosquitoes tested and number infected, respectively at location *s*_*i*_ and calendar month *t. Z*_*it*_ is assumed to arise from a binomial distribution, *Z*_*it*_~*Bin*(*N*_*it*_,*π*_*it*_) , where ***π***_*it*_ measures the SR at location *s*_*i*_ and time *t*. The regression function links the SR with other terms of the model (as shown for the density data) and is given as *logit* $logit(\pi_{it})=X^{T(2)}\beta^{\left(2\right)}+f{\left(t\right)}^{\left(2\right)}+U_{i}^{\left(2\right)}+{\mathrm{>\varepsilon}}_{t}^{\left(2\right)}+\varphi_{i}^{\left(2\right)}$. A similar specification described for the density model is followed in this model.

### Data management and environmental lags

To facilitate the assessment of the seasonal pattern, data were summarized by location and calendar month. That implies that all repeated surveys from a specific location within the same month were collapsed (sum of mosquito density/tested and positive) to a single observation.

To account for the environmental-lag-effect on mosquito density or SR, non-spatial (negative) binomial models (with/without zero inflation) were fitted and best lags were assessed. Lags refer to a climate/ environment value at different time intervals prior to the study date that might influence the amount of mosquitoes collected or the SR. Lags considered include the current month (month of collection of mosquitoes); 1/2/3 month(s) prior to the collection; average of current and one previous month; average of one and two previous months; and lastly average of current, one and two previous months. The analysis took into account seasonality, distance from water bodies and time (annual effect) which was incorporated as a binary variable indicating the year of study. Analysis was conducted separately for each species. Fitted values from models with all possible combinations of the environmental lags were calculated and plotted against the observed values (mosquito counts or SR). The combination which best fits the data was used for further analysis. This was implemented in STATA 10 (Stata Corps).

### Model validation and prediction

Models were fitted using a training set (85% of the data) randomly selected from the entire data. Validation of the model performance was done on the test locations (the remaining 15% of the data) The predictive ability of the model was assessed by specifically calculate different credible intervals with different probability coverage of the posterior predictive distribution and compare the percentage of test locations correctly predicted within these credible intervals (52). The best predictive ability of the model is observed when higher the number of test locations falls within the narrowest credible interval. The predicted power of the model at 95% credible interval is reported.

Using the estimates obtained from the models, SR and mosquito density were predicted for the whole Rufiji site. The prediction was done at the 250 m resolution.

### Calculation of EIR

The EIR can be estimated as a product of the SR and human-biting rate. Depending on the mosquito collection method used (human landing, light trap, etc.), the human-biting rate can be correctly approximated either by the number of blood meals taken on humans/mosquito/day or by the mosquito density. Established correlation between number of mosquitoes captured by light traps and human landing catches is usually used to adjust light trap collection to equivalence of biting catches and avoid collection bias (53). For this study, EIR was calculated as a product of SR and mosquito density and then adjusted using a correction factor of 1.605 to calibrate estimates obtained from light trap collection (28, 53, 54).

At a specific pixel *j* and month *t* the predicted values of SR, ${\hat{\pi }}_{jt}$ and mosquito density, ${\hat{\mu }}_{jt}$ were obtained for *A. funestus* and *A. gambiae* species. EIR estimates representing the infectious bite/person/day were calculated as:

$$\text{E}\hat{I}R_{jt}=1.605*\left(\left({\hat{\pi }}_{jt_{af}}*{\hat{\mu }}_{jt_{af}}\right)+\left({\hat{\pi }}_{jt_{ag}}*{\hat{\mu }}_{jt_{ag}}\right)\right)$$

where 1.605 is the correction factor.

The $E\hat{I}R_{jt}$ was then multiplied by 30.5 and 365 to obtain monthly and annual estimates, respectively. Monthly and annual maps were produced to show seasonal and temporal trends of the transmission.

### Geostatistical model implementation

The final model was implemented in OpenBUGS and parameters were estimated using the Gibbs sampler MCMC algorithm. The spatial variance parameter was sampled directly from its inverse gamma full conditional distributions using Gibbs sampling (39). The remaining parameters were simulated using Metropolis algorithm with a normal proposal distribution. The mean of the proposal distribution was the parameter estimated from the previous iteration with a fixed variance (55, 56). Two separate chains were run in parallel with a total of 150,000 iterations each. A burn-in of 20,000 iterations was done and the last 5,000 and 1,000 samples were used for posterior inference and prediction, respectively. The Gelman-Rubin model diagnostic tool (57) was used to assess convergence of chains before summarizing the results. The package ‘*fields*’ in R was used for selection of knots. For practical implementation of the geostatistical model 281 knots (2,479 unique locations) were selected for the density data (both species), 177 (415 unique locations) for SR analysis of *A. funestus* and 219 (639 unique locations) for SR of *A. gambiae*. Predictions and calculations of EIR were done in Fortran 95 (Compaq Visual Fortran Professional 6.6.0).

## Results

### Data description

**Fig. 1:**
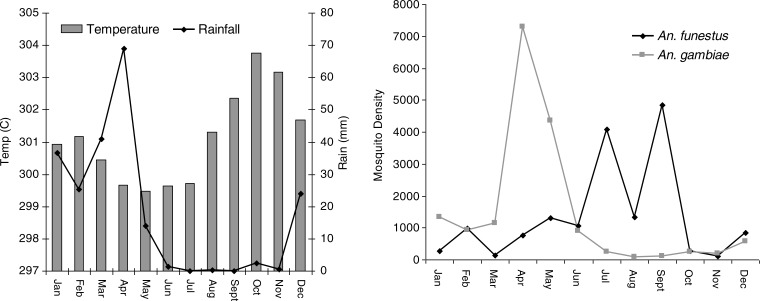
Seasonal variations of (A) rainfall, temperature and (B) mosquitoes densities of *A. gambiae* and *A. funestus* in the Rufiji DSS October 2001–September 2004.

In total of 2,479 unique locations were visited for the collection of the mosquitoes. A total of 15,983 *A. funestus* (from 18% of the surveyed locations, n=447) and 17,885 *A. gambiae* (from 27.3% of the surveyed locations, n=678) mosquitoes were captured. About 83 and 74.3% of the visits for mosquito collection for *A. funestus* and *A. gambiae* received zero counts. The crude annual SRs were 3.3, 2.8 and 3.2% for Year 1 (October 01–September 02), Year 2 (October 02–September 03) and Year 3 (October 03–September 04), respectively. The crude EIR were 507, 72.8 and 146 infectious bites/person/year for 3 years respectively. In Fig. 1, the relation between rainfall, temperature and mosquito density is shown (data collapsed in a period of one calendar year).

Most *A. gambiae* mosquitoes were captured during the months of April and May while most *A. funestus* were collected in the period of July–September. The number of *A. gambiae* collected was higher during the heavy rains while short rains with high temperature favour the population of *A. funestus* (Fig. 1).

### Geostatistical model results

Table 1 summarizes the results of parameter estimations from a multivariate geostatistical models on SRs and mosquito density.

**Table 1:** Results of association of environment/climate variables on sporozoite rate and mosquito density and spatio-temporal parameters

	Sporozoite rate		Density	
	
	Model: binomial		Model: zero inflated negative binomial	
	
Parameter	AF	AG	AF	AG
Seasonality	Median (95% CI^a^)		Median (95% CI^a^)	
Constant	0.04 (0.01, 0.23)	0.07 (0.02, 0.56)	1.03 (0.33, 2.4)	2.4 (0.53, 4.03)
Cos 12	0.99 (0.41, 2.41)	0.72 (0.29, 1.66)	1.1 (0.54, 2.3)	0.39 (0.2, 0.86)
Sin 12	0.84 (0.31, 2.53)	0.54 (0.19, 1.32)	0.75 (0.4, 1.55)	0.6 (0.32, 0.96)
Cos 6	1.27 (0.66, 2.47)	0.81 (0.44, 1.53)	0.75 (0.43, 1.39)	0.76 (0.41, 1.13)
Sin 6	0.65 (0.34, 1.25)	0.87 (0.45, 1.68)	1.13 (0.58, 2.08)	0.99 (0.53, 2.43)
Environment and climate				
NDVI	1.03 (0.85, 1.25)	0.93 (0.79, 1.1)	1.15 (0.87, 1.6)	1.11 (0.92, 1.35)
RAIN	0.96 (0.73, 1.26)	**0.53 (0.36, 0.79)**	**1.33 (1.06, 1.68)**	1.26 (0.97, 1.79)
LSTD	**2.31 (1.06, 6.97)**	0.92 (0.7, 1.22)	1.23 (0.81, 1.69)	**0.77 (0.64, 0.89)**
LSTN	1.04 (0.52, 3.51)	0.96 (0.73, 1.27)	**1.47 (1.02, 2.02)**	0.84 (0.69, 1.03)
Distance to the water bodies	0.93 (0.76, 1.11)	0.97 (0.85, 1.1)	0.96 (0.65, 1.22)	0.94 (0.79, 1.11)
Annual trend				
Year 2	1.01 (0.61, 1.67)	**0.48 (0.31, 0.75)**	**0.13 (0.08, 0.24)**	**0.17 (0.11, 0.25)**
Year 3	**0.41 (0.2, 0.79)**	**0.37 (0.24, 0.57)**	**0.34 (0.2, 0.61)**	**1.6 (1.04, 2.53)**
Spatial process				
Range^b^ (in km)^c^	35.52 (11.1, 78.81)	49.95 (15.54, 81.03)	21.1 (12.2, 56.6)	15.5 (8.9, 32.19)
Variance $\sigma_{sp}^{2}$	0.9 (0.37, 2.36)	0.45 (0.2, 1.18)	11.35 (6.58, 29.2)	5.04 (3.1, 10.33)
Temporal process				
Correlation ***γ***	0.5 (−0.52, 0.96)	0.5 (−0.51, 0.96)	−0.15 (−0.79, 0.67)	0.08 (−0.77, 0.83)
Variance $\sigma_{T}^{2}$	0.34 (0.14, 1.11)	0.33 (0.14, 0.94)	0.61 (0.22, 2.59)	0.51 (0.2, 2.55)
Other parameters				
Non-spatial variance $\sigma_{e}^{2}$	0.31 (0.16, 0.61)	0.34 (0.19, 0.59)	2.88 (1.81, 4.4)	2.59 (1.89, 3.2)
Over-dispersion ***r***	–	–	2.64 (1.7, 3.67)	1.16 (0.77, 1.61)
Covariates on the mixing probability				
Constant	–	–	0.07 (0.02, 0.21)	0.13 (0.06, 0.25)
NDVI	–	–	**0.3 (0.17, 0.54)**	0.93 (0.7, 1.29)
RAIN	–	–	1.3 (0.84, 5.37)	0.65 (0.36, 1.85)
LSTD	–	–	**0.07 (0.01, 0.64)**	**0.05 (0.02, 0.18)**
LSTN	–	–	0.53 (0.27, 1.14)	0.71 (0.28, 3.64)

a Credible Intervals (or posterior intervals).

b Based on spatial decay parameter, the Range is calculated as 3/**ρ** (×111 km).

c The spatial correlation is significant (>5%) within this distance.

Bold terms indicate significant variables in the model.

The effect of environmental variables differs significantly between species. Rain and temperature are the most influencing factors for density and sporozoite with higher effect on the *A. funestus* species. No significant effect of distance to the water bodies was obtained. highly pronounced with a significant decrease of mosquito population in Year 2 as compared to Year 1 and later an increase in the Year 3 as compared to Year 2. Spatial ranges are quite high especially for the SRs. The estimate of the over-dispersion parameter of *A. funestus* is twice as large as that of *A. gambiae* which could be influenced by the amount of zero counts in the data. However, the estimate of ***r*** is larger than 1 indicating that the data are not highly overdispersed (58). Day temperature significantly reduces the probability of observing zero mosquito counts. Spatial variability accounts more for the total variability in the data as compared to the non-spatial and temporal variability.

For a total of 63, 99, 368 and 368 test locations selected for validation of SR-AF, SR-AG, Density-AF and Density-AG models respectively, 68.3, 63.6, 84.1 and 89.9% of the locations were correctly predicted within 95% credible interval. Gelman-Rubin diagnostics indicated good convergence of all model parameters.

### Mapping of EIR

**Fig. 2:**
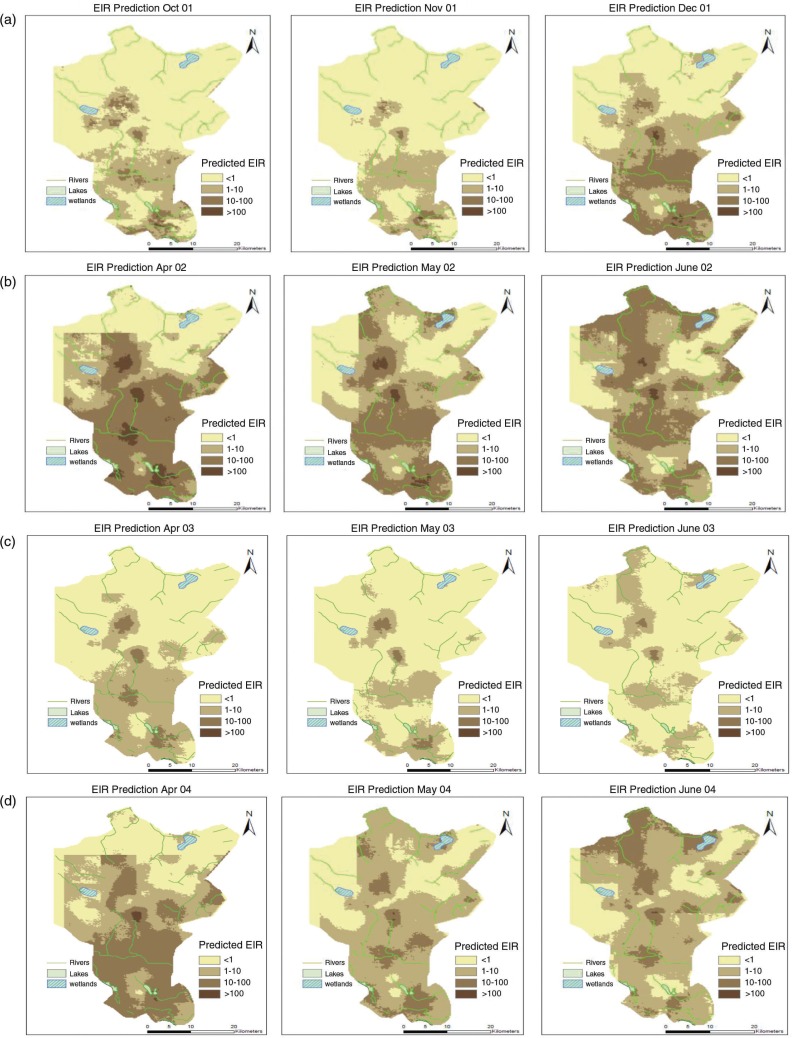
Selected EIR maps showing the spatial distribution and the seasonal pattern, for the period of Oct 2001–Sept 2004. (A) Dry months followed by the period of short rains, (B) Months immediately after the onset of heavy rains during the first year (very wet), (C) Months immediately after the onset of heavy rains during the second year (dry) and (D) Months immediately after the onset of heavy rain season during the third year (normal rains).

Figure 2 presents selected EIR maps for the Rufiji DSS site for the *A. funestus* and *A. gambiae*.

The southern part of the DSS showed high transmission throughout the years. High transmission was observed immediately at the onset of rains, especially during the heavy rain period. At the end of the rainy season (May–June), the transmission spread throughout the region (Fig. 2).

**Fig. 3:**
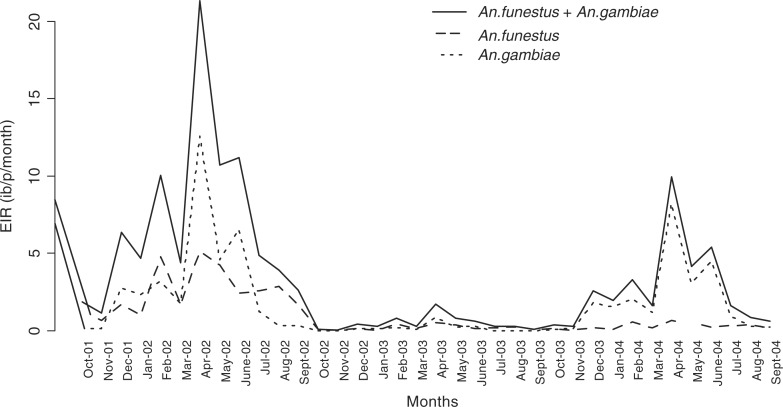
Predicted monthly EIR median and attribute of each species in Rufiji DSS.

In Fig. 3, monthly time series (median) predicted EIR are plotted for the entire study period. Attributes of each species are also indicated.

**Fig. 4:**
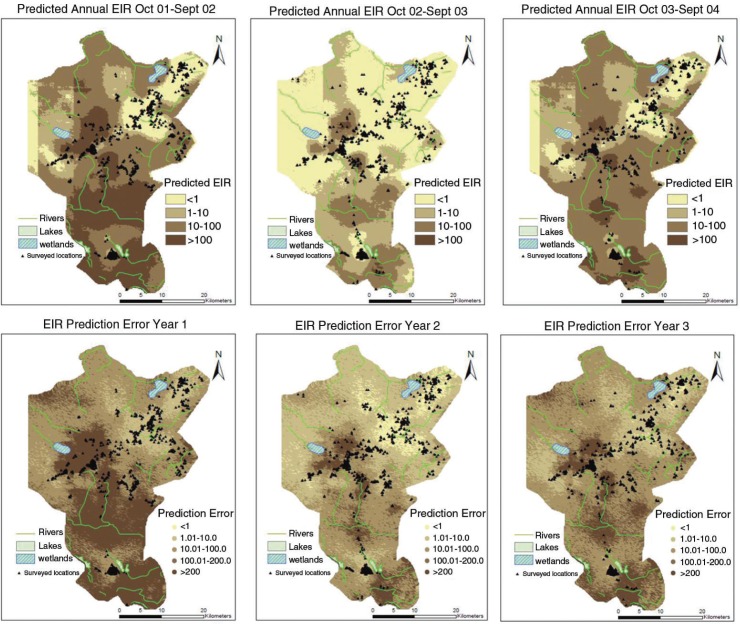
Spatial temporal distribution of annual EIR with prediction error maps.

The transmission starts peaking in the month of April (just after rains) and gradually drops in July (first year of the study). There was a reduction in the second year of the study and EIR increased again during the last year. A similar monthly trend is observed across years, which emphasizes seasonality. *A. funestus* are more prominent during the dry months while *A. gambiae* are more prominent during the rainy periods. The spatial temporal distribution of year-by-year EIR is shown in Fig. 4 with maps of prediction error. The prediction error for the EIR estimates was obtained my multiplying the prediction errors obtained from SR and density models.

Patterns in Fig. 4 show that few surveyed households are located in areas with EIR<1; however, a large proportion of household presented high transmission intensity. Higher prediction errors are seen in areas with few surveyed locations. The errors also capture the effect of heterogeneity arising from unmeasured factors.

### Population-adjusted EIR

**Fig. 5:**
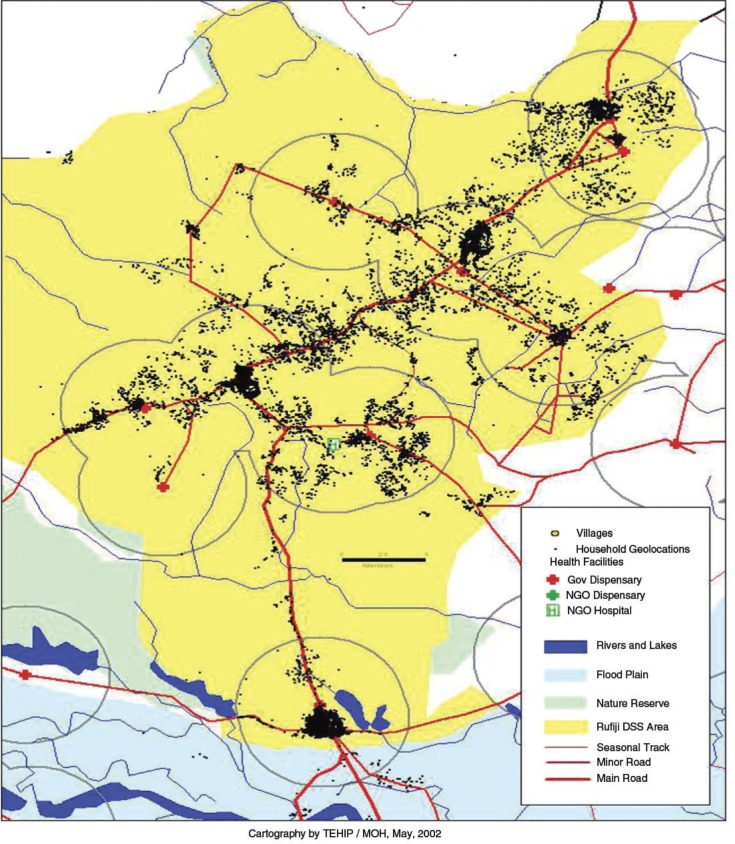
Distribution of households in the Rufiji DSS area (Source: TEHIP, 2002).

The annual and species-specific population-adjusted EIR were calculated by averaging predicted inoculation rates at all households (N=14,516) within the RDSS (Fig. 5) excluding all of the other pixels. Results are presented in Table 2.

**Table 2:** Overall predicted EIR with the percent attribute of each species

Period	*A. funestus*+*A. gambiae*	*A. funestus*	*A. gambiae*
Year 1	853.6	582.9 (68%)	270.7 (32%)
Year 2	113.7	88.8 (78%)	24.9 (22%)
Year 3	286.1	107.2 (37%)	178.9 (63%)

Overall transmission intensity reduced significantly during Year 2 and 3 as compared to Year 1 of the study. *A. funestus* was the main responsible vector for transmission in the first (68%) and second (78%) year, while the last year transmission was mainly driven by *A. gambiae* (63%).

In addition, we assessed the spatial shift (distribution) of transmission intensity over time, as illustrated in Table 3. EIR were categorized into five transmission intensities which were: no transmission (EIR=0), very low (EIR≥0.0–1), low (EIR≥1–10), average (EIR≥10–100), and high (EIR≥100). The change in the percentage of households exposed to a specific level of transmission was then studied.

**Table 3:** Distribution of predicted EIR over the RDSS area by Year, N* (%)

Category	EIR range	Year 1, *N*^a^(%)	Year 2, *N* (%)	Year 3, *N* (%)
No	0	4,896 (27.5)	13,124 (73.8)	4,225 (23.8)
Very low	>0.0–1	704 (4.0)	1,320 (7.4)	1,286 (7.2)
Low	>1–10	4,568 (25.7)	2,081 (11.7)	6,779 (38.1)
Average	>10–100	5,377 (30.2)	1,068 (6.0)	4,781 (26.9)
High	>100	2,238 (12.6)	190 (1.1)	712 (4.0)

a The number of households within a specific transmission intensities category.

The proportion of households predicted with very low transmission intensity increased between the first year and the third year of the study, from 4.0 to 7.2%. A significant reduction (over 68%) of locations with high transmission is seen during the last year of the study (i.e. 12.6% in the first year to 4% in the third year).

## Discussion

In this study, we assessed spatial–temporal variation and heterogeneity of malaria transmission in the Rufiji DSS site using a large geo-referenced biweekly entomological dataset collected over 3 years, and rigorous Bayesian geostatistical models. Our work is amongst the few to address spatial modelling of Entomology inoculation rate (EIR) based on sparse data by applying current Bayesian methodologies approximating spatial processes for large data. The INDEPTH-MTIMBA data, which was used in our application, is the most comprehensive entomological database in Africa. Bayesian spatio-temporal binomial and zero inflated negative binomial regression models were developed to produce monthly maps of EIR taking into account the malaria–climate relation and seasonality in transmission (35, 36, 59–61).

Geostatistical models have been widely used in malaria mapping in recent years (3, 38, 52, 62–64). Most of these analysis involved standard geostatistical models which are relevant for a moderate number of locations. Computation involved in these models is not feasible for data collected over a large number of survey locations. In this study, we used methods proposed by Barnejee et al. (40) and Finley et al. (42) to approximate the spatial process using a subset of survey locations selected via space filling design implemented in R software. Additive temporal correlations with autoregressive structure were also incorporated in all models. The predictive power of the model suggests good performance of the spatial correlation approximated from a subset of observed location. That might indicate that the subset selected was significantly appropriate. This work adds to the few in literature that indirect evaluates performance of using subsets to approximate the spatial process in real-life field data.

Changes in climate conditions, natural inhabitants and other human activities, which depend on the environment, alter the intensity of malaria transmission (21, 65). Our results depict temporal and seasonal variation in EIR along the study period and study area. Transmission was higher during the rainy periods with high temperatures and very low during the dry season or year. Two species *A. funestus* and *A. gambiae* are mainly responsible for malaria transmission in this region. Differences on the effect of environmental factors on the mosquito abundance and SRs of the species were observed. The population of *A. gambiae* increases at the onset of heavy rains while that of *A. funestus* peaks during the short rains season. Similar results have been reported in the Kilombero valley and other areas with similar climate in Africa and are associated with the preferential conditions of breeding sites of these species (13, 16, 66–70). A study, which assessed spatio-temporal variation of EIR in Navrongo DSS, showed similar patterns of seasonality that differed by species (28). Highly significant effects of temperature on the SR and density of *A. funestus* were observed. Contrary to *A. gambiae* which has relatively exophilic behaviour, this species is strictly endophilic, which could facilitate choice of conducive a resting environment favouring the gonotrophic cycle resulting in higher survival and hence longer infectivity (71–73). Knowledge of these characteristics can be important for understanding disease dynamics and for efficient implementation of interventions (5, 6, 66, 74).

There was considerable variation over short distances in the intensity of transmission. Small-scale variations in malaria transmission are common in sub-Saharan Africa and create complexity in implementing strategies to combat malaria (8, 28, 59, 75–77). The spatial correlation was still present over a substantial distance and the spatial variation comprised of about 90% of the total data variance. The spatial correlation arises partly due to spatial pattern in environmental drivers of transmission, partly due to effects of limited mosquito dispersion, and is also affected by human factors such as migration and human population densities (41, 42). We had an abundance of data on both mosquito and human populations; however, due to the relative small DSS area, it is difficult to separate the contributions of these different factors to the spatial correlation, which explains the higher spatial range. Such heterogeneity arising from unmeasured factors is captured by the prediction errors.

The methodology described in this study allows estimation of EIR while adjusting for both, temporal and small area spatial variations in a systematic and thorough manner. It acknowledges key characteristic of the data, considers computation difficulties and correlation among potential drivers of malaria transmission. It could be seen that the crude EIR were underestimated as compared to model-based estimations by over 55%. This underlines the importance of utilizing efficient methodologies while estimating epidemiological parameters to allow for proper decisions.

Our formulation allows further expansion and easy incorporation of other covariates in the main structure of the model either as specific covariates or their interaction. The complex component of our proposal is how to separately model SR and density data, incorporate seasonality, choosing environment lags and lastly approximate the spatial correlation when the large number of location has been observed. All these have been worked on. Moreover, DSS sites including Rufiji, collected comprehensive records of all-cause and disease mortality in the human population at the time of this entomological surveillance. The exposure surfaces estimated using this approach can be linked to mortality data to assess the malaria-specific mortality burden. Through that, much more accurate estimates of the benefits to be gained by reducing malaria transmission can be estimated than if it would have been possible from analyses that aggregate EIR over large areas and time periods or those fitted assuming normally distributed EIR.

## References

[1] WHO (2012). World malaria report.

[2] Hay SI, Snow RW (2006). The malaria atlas project: developing global maps of malaria risk. PLoS Med.

[3] Hay SI, Guerra CA, Gething PW, Patil AP, Tatem AJ, Noor AM (2009). A world malaria map: Plasmodium falciparum endemicity in 2007. PLoS Med.

[4] Parham PE, Michael E (2010). Modeling the effects of weather and climate change on malaria transmission. Environ Health Perspect.

[5] Fontenille D, Lochouarn L, Diatta M, Sokhna C, Dia I, Diagne N (1997). Four years’ entomological study of the transmission of seasonal malaria in Senegal and the bionomics of *Anopheles gambiae* and A. arabiensis. Trans R Soc Trop Med Hyg.

[6] Beier JC, Killeen GF, Githure JI (1999). Short report: entomologic inoculation rates and Plasmodium falciparum malaria prevalence in Africa. Am J Trop Med Hyg.

[7] Hay SI, Rogers DJ, Toomer JF, Snow RW (2000). Annual Plasmodium falciparum entomological inoculation rates (EIR) across Africa: literature survey, Internet access and review. Trans R Soc Trop Med Hyg.

[8] Mboera LEG, Senkoro KP, Mayala BK, Rumisha SF, Rwegoshora RT, Mlozi MRS (2010). Spatio-temporal variation in malaria transmission intensity in five agro-ecosystems in Mvomero district, Tanzania. Geospat Health.

[9] Nedelman J (1983). A negative binomial model for sampling mosquitoes in a malaria survey. Biometrics.

[10] Alexander N, Moyeed R, Stander J (2000). Spatial modelling of individual-level parasite counts using the negative binomial distribution. Biostatistics.

[11] Michael E (2001). Quantifying mosquito biting patterns on humans by DNA fingerprinting of bloodmeals. Am J Trop Med Hyg.

[12] Shaukat AM, Breman JG, McKenzie FE (2010). Using the entomological inoculation rate to assess the impact of vector control on malaria parasite transmission and elimination. Malar J.

[13] Smith T, Charlwood JD, Kihonda J, Mwankusye S, Billingsley P, Meuwissen J (1993). Absence of seasonal variation in malaria parasitaemia in an area of intense seasonal transmission. Acta Tropica.

[14] Killeen GF, McKenzie FE, Foy BD, Schieffelin C, Billingsley PF, Beier JC (2000). A simplified model for predicting malaria entomologic inoculation rates based on entomologic and parasitologic parameters relevant to control. Am J Trop Med Hyg.

[15] Lee HI, Lee JS, Shin EH, Lee WJ, Kim YY, Lee KR (2001). Malaria transmission potential by *Anopheles sinensis* in the Republic of Korea. Korean J Parasitol.

[16] Kelly-Hope LA, McKenzie FE (2009). The multiplicity of malaria transmission: a review of entomological inoculation rate measurements and methods across sub-Saharan Africa. Malar J.

[17] Snow RW, Craig MH, Deichmann U, le Sueur D (1999). A preliminary continental risk map for malaria mortality among African children. Parasitol Today (Regul Ed).

[18] Woolhouse MEJ, Dye C, Etard J-F, Smith T, Charlwood JD, Garnett GP (1997). Heterogeneities in the transmission of infectious agents: implications for the design of control programs. Proc Natl Acad Sci U S A.

[19] Brogan R, Zhao C (1992). Designing a longitudinal study [microform]: issues, problems & concerns. [S.l.]: distributed by ERIC Clearinghouse. http://www.eric.ed.gov/contentdelivery/servlet/ERICServlet?accno=ED401316.

[20] Smith TA, Leuenberger R, Lengeler C (2001). Child mortality and malaria transmission intensity in Africa. Trends Parasitol.

[21] Thomson MC, Connor SJ (2001). The development of malaria early warning systems for Africa. Trends Parasitol.

[22] Sankoh OA, Binka F, Takken W, Martens P, Bogers RJ (2005). INDEPTH Network: a viable platform for the assessment of malaria risk in developing countries. Environmental change and malaria risk-global and local implications.

[23] Gatton M (2010). Environmental factors and malaria transmission risk. Lancet Infect Dis.

[24] Killeen GF, Tami A, Kihonda J, Okumu FO, Kotas ME, Grundmann H (2007). Cost-sharing strategies combining targeted public subsidies with private-sector delivery achieve high bednet coverage and reduced malaria transmission in Kilombero Valley, southern Tanzania. BMC Infect Dis.

[25] Leisnham PT, Slaney DP, Lester PJ, Weinstein P, Heath ACG (2007). Mosquito density, macroinvertebrate diversity, and water chemistry in water-filled containers: relationships to land use. New Zeal J Zool.

[26] Chase JM, Shulman RS (2009). Wetland isolation facilitates larval mosquito density through the reduction of predators. Ecol Entomol.

[27] Kweka EJ, Mwang'onde BJ, Mahande AM (2010). Optimization of odour-baited resting boxes for sampling malaria vector, Anopheles arabiensis Patton, in arid and highland areas of Africa. Parasit Vectors.

[28] Kasasa S, Asoala V, Gosoniu L, Anto F, Adjuik M, Tindana C (2013). Spatio-temporal malaria transmission patterns in Navrongo demographic surveillance site, northern Ghana. Malar J.

[29] Greene WH (1994). Accounting for excess zeros and sample selection in Poisson and negative binomial regression models.

[30] Cheung YB (2002). Zero-inflated models for regression analysis of count data: a study of growth and development. Stat Med.

[31] Yau KKW, Wang K, Lee AH (2003). Zero-inflated negative binomial mixed regression modeling of over-dispersed count data with extra zeros. Biometrical J.

[32] Ryan PA, Lyons SA, Alsemgeest D, Thomas P, Kay BH (2004). Spatial statistical analysis of adult mosquito (Diptera: Culicidae) counts: an example using light trap data, in Redland Shire, Southeastern Queensland, Australia. J Med Entomol.

[33] Ridout M, Hinde J, DeméAtrio CGB (2001). A score test for testing a zero-inflated Poisson regression model against zero-inflated negative binomial alternatives. Biometrics.

[34] Agarwal DK, Gelfand AE, Citron-Pousty S (2002). Zero-inflated models with application to spatial count data. Environ Ecol Stat.

[35] Warton DI (2005). Many zeros does not mean zero inflation: comparing the goodness-of-fit of parametric models to multivariate abundance data. Environmetrics.

[36] Sogoba N, Vounatsou P, Doumbia S, Bagayoko M, Touré MB, Sissoko IM (2007). Spatial analysis of malaria transmission parameters in the rice cultivation area of Office du Niger, Mali. Am J Trop Med Hyg.

[37] Cressie NAC (1993). Statistics for spatial data.

[38] Diggle PJ, Tawn JA, Moyeed RA (1998). Model-based geostatistics. J Roy Stat Soc C Appl Stat.

[39] Gelfand AE, Smith AFM (1990). Sampling-based approaches to calculating marginal densities. J Am Stat Assoc.

[40] Banerjee S, Gelfand AE, Finley AO, Sang H (2008). Gaussian predictive process models for large spatial data sets. J R Stat Soc Series B Stat Methodol.

[41] Eidsvik J, Finley AO, Banerjee S, Rue H (2012). Approximate Bayesian inference for large spatial datasets using predictive process models. Comput Stat Data Anal.

[42] Finley AO, Sang H, Banerjee S, Gelfand AE (2009). Improving the performance of predictive process modeling for large datasets. Comput Stat Data Anal.

[43] Rumisha SF (2013). Modelling the seasonal and spatial variation of malaria transmission in relation to mortality in Africa.

[44] Mwageni E, Momburi D, Juma Z, Irema M, Masanja H, TEHIP and AMMP Teams (2002). INDEPTH monograph series: demographic surveillance systems for assessing populations and their health in developing countries.

[45] Stolwijk AM, Straatman H, Zielhuis GA (1999). Studying seasonality by using sine and cosine functions in regression analysis. J Epidemiol Community Health.

[46] Rau R (2006). Seasonality in human mortality – a demographic approach. http://www.springer.com/economics/population/book/978-3-540-44900-3.

[47] Lambert D (1992). Zero-inflated Poisson regression, with an application to defects in manufacturing. Technometrics.

[48] Seeger M (2003). Bayesian Gaussian Process Models: PAC-Bayesian generalisation error bounds and sparse approximations. http://www.era.lib.ed.ac.uk/handle/1842/321.

[49] Xia G, Gelfand AE (2005). Stationary process approximation for the analysis of large spatial datasets. Technical Report.

[50] Johnson ME, Moore LM, Ylvisaker D (1990). Minimax and maximin distance designs. J Stat Plann Infer.

[51] Hay JL, Pettitt AN (2001). Bayesian analysis of a time series of counts with covariates: an application to the control of an infectious disease. Biostatistics.

[52] Gosoniu L, Vounatsou P, Sogoba N, Smith T (2006). Bayesian modelling of geostatistical malaria risk data. Geospat Health.

[53] Lines JD, Wilkes TJ, Lyimo EO (1991). Human malaria infectiousness measured by age-specific sporozoite rates in *Anopheles gambiae* in Tanzania. Parasitology.

[54] Amek N, Bayoh N, Hamel M, Lindblade KA, Gimnig JE, Odhiambo F (2012). Spatial and temporal dynamics of malaria transmission in rural Western Kenya. Parasites & Vectors.

[55] Hastings WK (1970). Monte Carlo sampling methods using Markov chains and their applications. Biometrika.

[56] Metropolis N (1987). The beginning of the Monte Carlo method. Los Alamos Science.

[57] Gelman A, Rubin DB (1992). Inference from iterative simulation using multiple sequences. Statist Sci.

[58] Lloyd-Smith JO (2007). Maximum likelihood estimation of the negative binomial dispersion parameter for highly overdispersed data, with applications to infectious diseases. PLoS One.

[59] Thomas CJ, Lindsay SW (2000). Local-scale variation in malaria infection amongst rural Gambian children estimated by satellite remote sensing. Trans R Soc Trop Med Hyg.

[60] Gosoniu L (2008). Development of Bayesian geostatistical models with applications in malaria epidemiology [doctoral]. http://ihi.eprints.org/1131/.

[61] Reid H, Vallely A, Taleo G, Tatem AJ, Kelly G, Riley I (2010). Baseline spatial distribution of malaria prior to an elimination programme in Vanuatu. Malar J.

[62] Amek N, Bayoh N, Hamel M, Lindblade KA, Gimnig J, Laserson KF (2011). Spatio-temporal modeling of sparse geostatistical malaria sporozoite rate data using a zero inflated binomial model. Spat Spatiotemporal Epidemiol.

[63] Gething PW, Patil AP, Smith DL, Guerra CA, Elyazar IRF, Johnston GL (2011). A new world malaria map: Plasmodium falciparum endemicity in 2010. Malar J.

[64] Giardina F, Gosoniu L, Konate L, Diouf MB, Perry R, Gaye O (2012). Estimating the Burden of Malaria in Senegal: Bayesian Zero-Inflated Binomial Geostatistical Modeling of the MIS 2008 Data. PLoS ONE.

[65] Snow RW, Nahlen B, Palmer A, Donnelly CA, Gupta S, Marsh K (1998). Risk of severe malaria among African infants: direct evidence of clinical protection during early infancy. J Infect Dis.

[66] Gillies MT, de Meillon B (1968). The Anophelinae of Africa south of the Sahara (Ethiopian Zoogeographical Region). Publ S Afr Inst Med Res.

[67] Charlwood JD, Vij R, Billingsley PF (2000). Dry season refugia of malaria-transmitting mosquitoes in a dry savannah zone of east Africa. Am J Trop Med Hyg.

[68] Warrell DA, Gilles HM (2003). Essential malariology.

[69] Guelbeogo WM, Sagnon N, Grushko O, Yameogo MA, Boccolini D, Besansky NJ (2009). Seasonal distribution of *Anopheles funestus* chromosomal forms from Burkina Faso. Malar J.

[70] Adja AM, N'goran EK, Koudou BG, Dia I, Kengne P, Fontenille D (2011). Contribution of *Anopheles funestus, An. gambiae and An. nili* (Diptera: Culicidae) to the perennial malaria transmission in the southern and western forest areas of Côte d'Ivoire. Ann Trop Med Parasitol.

[71] Charlwood JD, Qassim M, Elnsur EI, Donnelly M, Petrarca V, Billingsley PF (2001). The impact of indoor residual spraying with malathion on malaria in refugee camps in eastern Sudan. Acta Trop.

[72] Kent RJ, Coetzee M, Mharakurwa S, Norris DE (2006). Feeding and indoor resting behaviour of the mosquito Anopheles longipalpis in an area of hyperendemic malaria transmission in southern Zambia. Med Vet Entomol.

[73] Atieli H, Menya D, Githeko A, Scott T (2009). House design modifications reduce indoor resting malaria vector densities in rice irrigation scheme area in western Kenya. Malar J.

[74] Thomson MC, Mason SJ, Phindela T, Connor SJ (2005). Use of rainfall and sea surface temperature monitoring for malaria early warning in Botswana. Am J Trop Med Hyg.

[75] Drakeley CJ, Carneiro I, Reyburn H, Malima R, Lusingu JPA, Cox J (2005). Altitude-dependent and -independent variations in Plasmodium falciparum prevalence in northeastern Tanzania. J Infect Dis.

[76] Stewart L, Gosling R, Griffin J, Gesase S, Campo J, Hashim R (2009). Rapid assessment of malaria transmission using age-specific sero-conversion rates. PLoS ONE.

[77] Bousema T, Okell L, Shekalaghe S, Griffin JT, Omar S, Sawa P (2010). Revisiting the circulation time of Plasmodium falciparum gametocytes: molecular detection methods to estimate the duration of gametocyte carriage and the effect of gametocytocidal drugs. Malar J.

